# Lack of impact of the ALDH2 rs671 variant on breast cancer development in Japanese BRCA1/2‐mutation carriers

**DOI:** 10.1002/cam4.5430

**Published:** 2022-11-07

**Authors:** Tomoharu Mori, Yusuke Okamoto, Anfeng Mu, Yoshimi Ide, Akiyo Yoshimura, Noriko Senda, Yukiko Inagaki‐Kawata, Masahiro Kawashima, Hiroyuki Kitao, Eriko Tokunaga, Yasuo Miyoshi, Shozo Ohsumi, Koichiro Tsugawa, Tomohiko Ohta, Toyomasa Katagiri, Shigeru Ohtsuru, Kaoru Koike, Seishi Ogawa, Masakazu Toi, Hiroji Iwata, Seigo Nakamura, Keitaro Matsuo, Minoru Takata

**Affiliations:** ^1^ Laboratory of DNA Damage Signaling, Department of Late Effects Studies Radiation Biology Center Graduate School of Biostudies, Kyoto University Kyoto Japan; ^2^ Department of Primary Care and Emergency Medicine Graduate School of Medicine, Kyoto University Kyoto Japan; ^3^ Division of Breast Surgical Oncology Showa University School of Medicine Tokyo Japan; ^4^ Department of Breast Surgery Kikuna Memorial Hospital Yokohama Japan; ^5^ Department of Breast Oncology Aichi Cancer Center Hospital Nagoya Japan; ^6^ Department of Breast Surgery Graduate School of Medicine Kyoto University Kyoto Japan; ^7^ Department of Molecular Cancer Biology Graduate School of Pharmaceutical Sciences, Kyushu University Fukuoka Japan; ^8^ Department of Breast Oncology National Hospital Organization Kyushu Cancer Center Fukuoka Japan; ^9^ Division of Breast and Endocrine Surgery Department of Surgery, Hyogo College of Medicine Hyogo Japan; ^10^ Department of Breast Oncology National Hospital Organization Shikoku Cancer Center Matsuyama Ehime Japan; ^11^ Division of Breast and Endocrine Surgery, Department of Surgery St. Marianna University School of Medicine Kawasaki Kanagawa Japan; ^12^ Department of Translational Oncology St. Marianna University Graduate School of Medicine Kawasaki Kanagawa Japan; ^13^ Division of Genome Medicine Institute of Advanced Medical Sciences Tokushima University Tokushima Japan; ^14^ Department of Pathology and Tumor Biology Graduate School of Medicine Kyoto University Kyoto Japan; ^15^ Department of Medicine Center for Hematology and Regenerative Medicine Karolinska Institute Solna Sweden; ^16^ Institute for the Advanced Study of Human Biology (WPI‐ASHBi) Kyoto University Kyoto Japan; ^17^ Division of Cancer Epidemiology and Prevention Aichi Cancer Center Research Institute Nagoya Aichi Japan; ^18^ Division of Cancer Epidemiology Nagoya University Graduate School of Medicine Nagoya Aichi Japan; ^19^ Present address: Lunenfeld‐Tanenbaum Research Institute, Mount Sinai Hospital Toronto Ontario Canada

**Keywords:** Fanconi anemia, hereditary breast and ovarian cancer, BRCA1, BRCA2, ALDH2

## Abstract

The aldehyde degrading function of the ALDH2 enzyme is impaired by Glu504Lys polymorphisms (rs671, termed A allele), which causes alcohol flushing in east Asians, and elevates the risk of esophageal cancer among habitual drinkers. Recent studies suggested that the *ALDH2* variant may lead to higher levels of DNA damage caused by endogenously generated aldehydes. This can be a threat to genome stability and/or cell viability in a synthetic manner in DNA repair‐defective settings such as Fanconi anemia (FA). FA is an inherited bone marrow failure syndrome caused by defects in any one of so far identified 22 *FANC* genes including hereditary breast and ovarian cancer (HBOC) genes *BRCA1* and *BRCA2*. We have previously reported that the progression of FA phenotypes is accelerated with the *ALDH2* rs671 genotype. Individuals with HBOC are heterozygously mutated in either *BRCA1* or *BRCA2*, and the cancer‐initiating cells in these patients usually undergo loss of the wild‐type *BRCA1/2* allele, leading to homologous recombination defects. Therefore, we hypothesized that the *ALDH2* genotypes may impact breast cancer development in *BRCA1/2* mutant carriers. We genotyped *ALDH2* in 103 HBOC patients recruited from multiple cancer centers in Japan. However, we were not able to detect any significant differences in clinical stages, histopathological classification, or age at clinical diagnosis across the *ALDH2* genotypes. Unlike the effects in hematopoietic cells of FA, our current data suggest that there is no impact of the loss of *ALDH2* function in cancer initiation and development in breast epithelium of HBOC patients.

## Graphical abstract

The common ALDH2 variant rs671 severely aggravates DNA repair‐deficient disorder Fanconi anemia, and it is expected that breast cancer developed in BRCA1/2 mutation carriers are similarly affected. Now we elucidated this is not the case in the actual patient cohort, highlighting cell type‐specific differences in the protection/detoxification mediated by ALDH2.

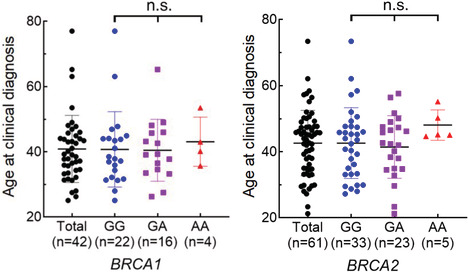

## 1 INTRODUCTION

The aldehyde degrading function of the ALDH2 enzyme is impaired by the Glu504Lys polymorphisms (rs671, termed A allele). This variant is prevalent among East Asian countries, and about 40% or 10% of the Japanese population carries rs671 in a heterozygous or homozygous manner, respectively. The rs671 causes alcohol flushing in East Asians, and elevates risks of esophageal cancer among habitual drinkers.^1^, ^2^, ^3^ Recent studies have suggested that the *ALDH2* variant can lead to higher levels of DNA damage caused by aldehydes such as acetaldehyde generated by ethanol metabolism. It is now becoming clearer that DNA damaging aldehydes do not necessarily derive from exogenous sources but also come from endogenous metabolic activities (i.e., formaldehyde generated by histone demethylation reactions).^4^, ^5^ Such endogenous aldehydes are well tolerated but can be a threat to genome stability and/or cell viability in DNA repair‐defective settings in a synthetic manner.^6^, ^7^ One example is the DNA repair‐deficient disorder Fanconi anemia with the *ALDH2* variant. FA is the most common form among the inherited bone marrow failure syndromes (IBMFS), which is caused by defects in any one of so far identified 22 *FANC* genes with clinical features including leukemia/myelodysplasia and solid tumors.^8^ Strikingly, when FA patients carry the homozygous *ALDH2* variant (AA genotype), they invariably display a severe phenotype immediately after birth, such as hypoplastic anemia as well as myelodysplastic syndrome (MDS), reflecting loss of hematopoietic stem cells or accumulating mutations.^9^ The GA genotype also accelerates the progression of bone marrow failure.^9^

Germline mutations in the *BRCA1* or *BRCA2* gene, which are critical regulators of RAD51, a key player in homologous recombination (HR), predispose women to breast or ovarian cancer.^10^ In patients with hereditary breast and ovarian cancer (HBOC) syndrome, who are heterozygously mutated in either *BRCA1* or *BRCA2*, cancer‐initiating cells mostly undergo loss of wild‐type *BRCA1/2* allele.^11^ Thus, tumors developed in HBOC patients are defective in HR (termed HRD or BRCAness).^12^ Notably, biallelic *BRCA1* and *BRCA2* mutations are found in a subset of FA cases known as the complementation group of FA‐S and FA‐D1, respectively.^8^ Interestingly, it has been reported that *ALDH2* activity is required for maintaining genome stability and/or cell viability in HRD tumors or leukemia in a synthetic manner.^6^, ^7^ These notions raised a hypothesis that cancer development in HBOC patients might be retarded or abrogated by the loss or decrease of *ALDH2* function in cases with AA or GA genotypes, respectively. Alternatively, if HBOC cancer is developed analogously to the situation in FA, in which leukemia/MDS development was accelerated with the AA but not with GA genotype,^9^ HBOC cancer may appear earlier in the AA cases.

Another study found that exogenously added aldehydes destabilize the BRCA2 protein.^13^ Thus, they hypothesized that increased levels of endogenously generated acetaldehyde can be the basis for haploinsufficiency of the *BRCA2* gene in preventing carcinogenesis.^13^ In this scenario, cells in *ALDH2* defective individuals may have destabilized BRCA2 proteins, forming the basis for haploinsufficiency and accelerated cancer development.

Thus, we collected clinicopathological information from Japanese *BRCA1/2* mutation carriers and genotyped *ALDH2* in them. On the contrary to the expectation from these prior studies, we could not prove the significant impact of *ALDH2* genotype on cancer development among our cohort of patients.

## 2 MATERIALS AND METHODS

### 2.1 Patients and saliva samples

The research plan was registered at the UMIN Clinical Trial Registry (https://www.umin.ac.jp/ctr/index‐j.htm) with the ID number UMIN000030137 and approved by the Ethical Committee of Kyoto University and the other participating institutions/hospitals. Written informed consent was obtained from all recruited subjects. Information regarding identified *BRCA* mutations, clinical and histopathological features, cancer cell phenotypes, or drinking status was recorded using a survey form. Clinical staging, pathological classification, and ER, PgR, HER2 status were evaluated in each participating breast cancer center with established methods, and the data were collected using the standardized format. These data were described according to the General rules for clinical and pathological recording of breast cancer published by the Japanese Breast Cancer Society.^18^ *BRCA* genetic testing was performed in each cancer center according to the guidelines set by Japan Registered Clinical Laboratories Association, The Ministry of Health, Labour and Welfare of Japan, The Japanese Association of Medical Sciences, and The Japanese Society for Hereditary Tumors. Samples were sent to Myriad Genetic Laboratories (Salt Lake City, USA) via FALCO Biosystems (Kyoto, https://www.falco.co.jp/) after obtaining full individual informed consent for the BRCA testing. Detected variants were classified according to the criteria of Myriad. In some cases, *BRCA* testing was done by next‐generation sequencing and judged as in ClinVar.^11^ Saliva samples were collected using an Oragene kit (DNA Genotek Inc) and genomic DNA was isolated according to the manufacturer's instructions.

### 2.2 *ALDH2* genotyping

*ALDH2* genotyping was carried out with Taqman PCR as previously described.^9^, ^14^ Briefly, PCR reaction was carried out with ~50 ng of template DNA, 2× TaqMan Universal Master Mix buffer (Applied Biosystems), and 20× primer and probe Mix (Applied Biosystems) using CFX96 Real‐time System (BIO‐RAD).

### 2.3 Cell culture and growth assay

HCT116 and MCF7 cells were maintained in McCoy's 5A (Sigma) and IMDM (Nacalai Tesque), respectively, each supplemented with 10% FBS (Gibco). Cells were treated with indicated doses of disulfiram (TCI) and/or B02 (Selleckchem) for 3 days and cell growth in a 96‐well plate was measured using an MTA cell proliferation kit (Nacalai Tesque). Statistical analyses were carried out using Prism software (Graphpad).

## 3 RESULTS

To observe potential genetic interactions between *BRCA1/2* and *ALDH2* in a real‐world setting, we set out to recruit cases of *BRCA1* and *BRCA2* mutation carriers from breast cancer centers in Japan, and we determined *ALDH2* genotypes using the previously established Taqman PCR assay. Assuming a 5‐year difference in the age of cancer development between *ALDH2* GG and GA/AA genotypes, we estimated that 100 cases with the *BRCA1* or *BRCA2* mutation would be necessary to detect the difference with an acceptable statistical power (expected mean age 55 in GG, 50 in GA/AA with standard deviation 10 with alpha error (0.05) and beta error (0.30)). Saliva samples, the results of *BRCA* gene testing, and clinical information (cancer staging, histopathological classification, and tumor subtypes as defined by expression of estrogen receptor (ER), progesterone receptor (PgR), and HER2 including drinking status (using a questionnaire)) were collected. Our recruitment criteria excluded cases of non‐BRCA mutations and variants of unknown significance (VUS). In total, 112 cases including one male were recruited from the breast cancer centers in Japan during the study period (2018 to 2020). One recruited case was mutated in *PALB2* and excluded. 7 cases that could not be genotyped because of poor sample quality were also excluded (Table 1). Data from the 103 cases (all females) were subjected to the analysis.

**TABLE 1:** Characteristics of the patients enrolled in this study

	Patient numbers
Onset of breast cancer	
Yes	101
No	2
BRCA variant carriers	
*BRCA1*	42
*BRCA2*	61
Age at cancer diagnosis	
20–29	13
30–39	29
40–49	43
50–59	11
60–69	3
70≦	2
Distribution of BRCA1/2 variants	
*BRCA1*	
Missense	13
Nonsense	9
InDel	19
Duplication	1
*BRCA2*	
Missense	13
Nonsense	17
InDel	30
Duplication	1
Histology	
Noninvasive carcinoma	5
Invasive carcinoma	
Invasive ductal carcinoma	82
Special types^a^	6
Unknown	8
Tumor subtypes	
*BRCA1*	
Luminal‐like (ER+/Her2‐)	11
Luminal‐like/Her2+ (ER+/Her2+)	0
TN (ER‐/Her2‐)	24
Pure Her2 (ER‐/Her2+)	0
Unknown	6
*BRCA2*	
Luminal‐like (ER+/Her2‐)	27
Luminal‐like/Her2+ (ER+/Her2+)	5
TN (ER‐/Her2‐)	11
Pure Her2 (ER‐/Her2+)	1
Unknown	16
Clinical stage of breast cancer	
*BRCA1*	
Unaffected	1
0	0
I	14
II	20
III	4
IV	0
Unknown	3
*BRCA2*	
Unaffected	1
0	3
I	17
II	23
III	11
IV	1
Unknown	5
Drinking history	
Yes	
Once a week≦	50
<Once a week	5
No	48

^a^ Medullary carcinoma, invasive lobular carcinoma, matrix‐producing carcinoma, metaplastic carcinoma, spindle cell carcinoma.

The characteristics of the patient cohort are summarized in Table 1, and detailed information from each case is provided in Tables S1 and S2. All of them had mutations classified as pathological (Table S1). 101 cases already developed breast cancer and three of them also had ovarian cancer. The distribution of *ALDH2* genotypes in both *BRCA1* (*n* = 42) or *BRCA2* (*n* = 61) mutated cases appeared not to skew significantly from those of the general Japanese population^1^ and therefore did not disrupt the Hardy–Weinberg equilibrium (Table 2). This essentially excluded the possibility that *ALDH2* deficiency drastically abrogates or accelerates the cancer development in HBOC patients.

**TABLE 2:** *BRCA* and *ALDH2* genotype of the patients

Mutated *BRCA* gene	*ALDH2* genotype	# of cases (%)	Type of *BRCA* mutation			
			Missense	Nonsense	InDel	Dup
*BRCA1*	Total (%)	42 (100)	13 (31.0)	9 (21.4)	19 (45.2)	1 (2.4)
	GG	22 (52.4)	8	7	7	0
	GA	16 (38.1)	4	1	10	1
	AA	4 (9.5)	1	1	2	0
*BRCA2*	Total (%)	61 (100)	13 (21.3)	17 (27.9)	30 (49.2)	1 (1.6)
	GG	33 (54.1)	6	9	18	0
	GA	23 (37.7)	6	7	9	1
	AA	5 (8.2)	1	1	3	0

**FIGURE 1:**
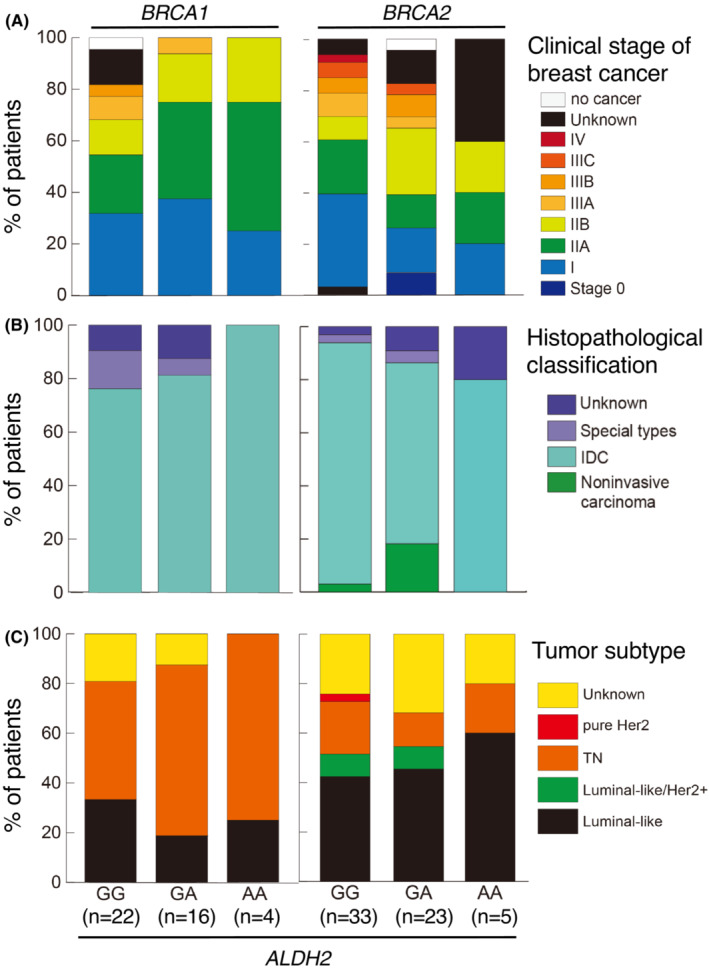
Characteristics of breast cancer in the *BRCA1* or *BRCA2* mutation carriers with different *ALDH2* genotypes. (A) Clinical stage of breast tumor in *BRCA1*‐ or *BRCA2‐*mutated patients. (B) Pathological classification of breast tumor in *BRCA1*‐ or *BRCA2‐*mutated patients. IDC, invasive ductal carcinoma. (C) Subtype of breast tumor in *BRCA1*‐ or *BRCA2‐*mutated patients. Triple negative (TN) tumors were defined by the results of ER and HER2 staining irrespective of PgR staining. In metachronous tumors, only the initial tumor was classified. In the case with simultaneously diagnosed bilateral tumors, more progressed tumor was included in the analysis.

We looked at the clinical staging, pathological features, and cancer cell phenotypes. There seemed to be no notable differences across *ALDH2* genotypes (Figure 1A–C). However, we noted that *BRCA*1 mutated cases displayed a higher incidence of triple‐negative (TN) breast cancer than in *BRCA2* cases, regardless of *ALDH2* genotypes (Figure 1C). This is in line with a previous observation^15^ and suggests that our patient cohort did not grossly deviate from the general HBOC population.

**FIGURE 2:**
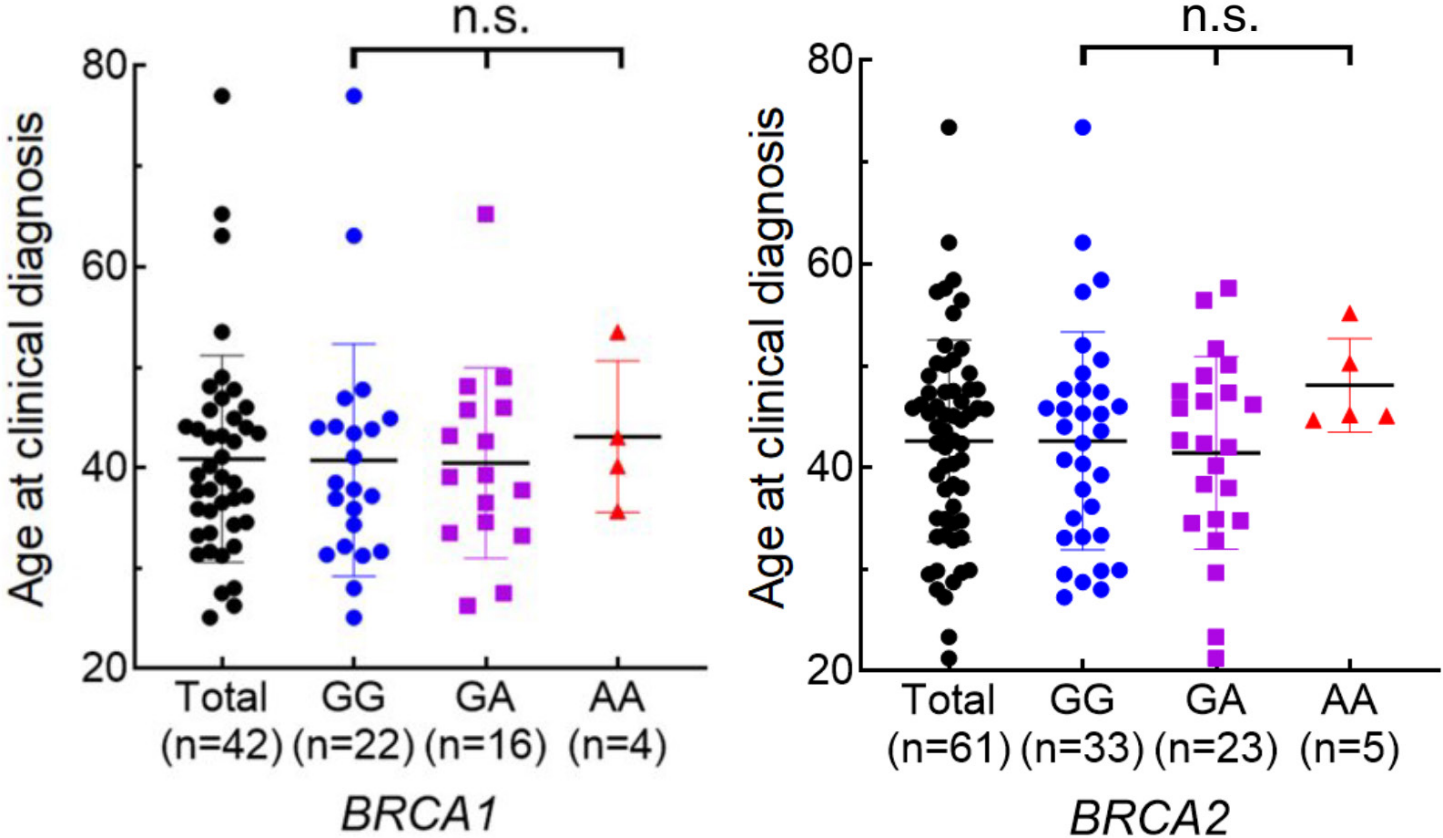
Effects of *ALDH2* genotypes on cancer development. Age at clinical diagnosis of breast cancer with indicated *BRCA* and *ALDH2* genotypes. Mean and SD are shown. n.s., statistically non‐significant as determined by one‐way ANOVA test. For metachronous tumors, the age at the initial tumor diagnosis was shown.

Next, to observe the effects of the *ALDH2* genotype on breast cancer initiation/development, we compared the age at clinical diagnosis in each *ALDH2* subgroup. The ALDH2 enzymatic activity is strongly reduced in the GA (lowered to ~10% because of the dominant‐negative effects) or in the AA (essentially null) genotypes. In both *BRCA1* and *BRCA2* mutated cohorts, the mean age at cancer diagnosis is not statistically different in cases with *ALDH2* AA genotype than those with the GG or GA (Figure 2A). We also note no significant difference between GG vs combined GA/AA subgroups. At this moment, we could not exclude the possibility that an analysis of a higher number of patients may show statistically significant effects. In any case, the impact seems marginal.

**FIGURE 3:**
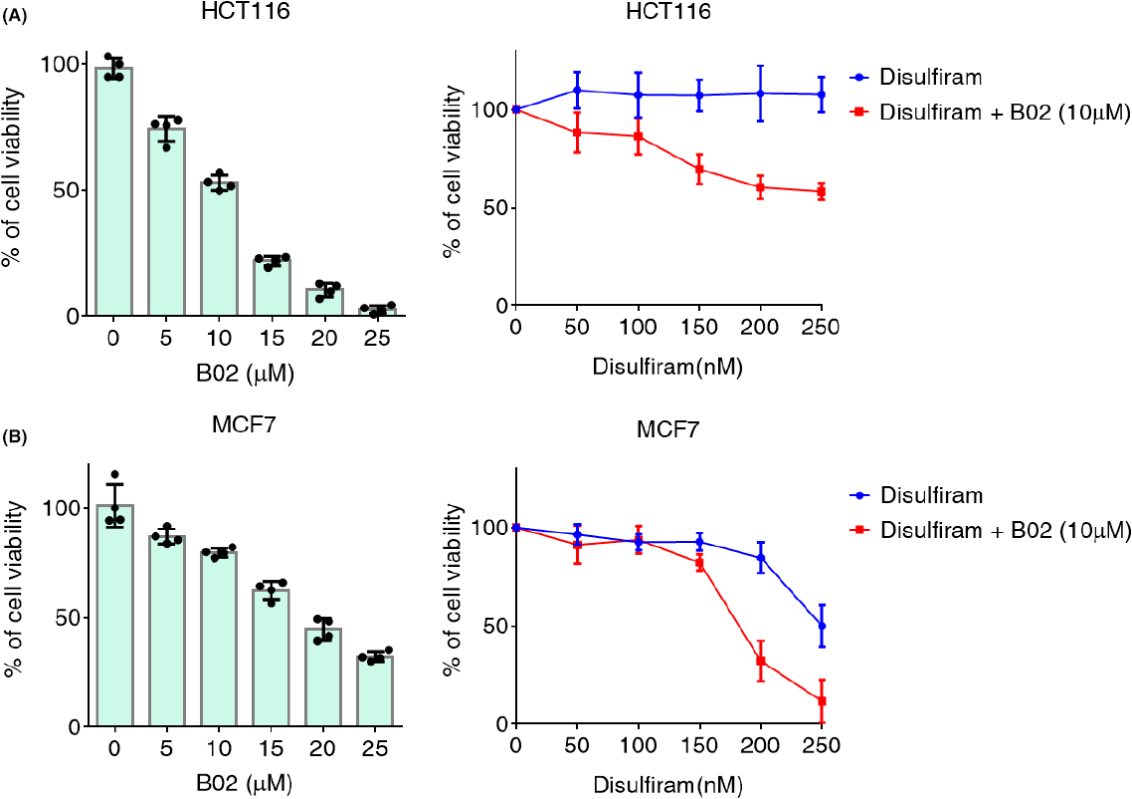
Effects of ALDH2 and RAD51 inhibition on cell growth. Effects of ALDH2 inhibitor disulfiram on the growth of cancer cell lines treated with RAD51 inhibitor B02. HCT116 (A) or MCF7 (B) cells were treated with B02 (left) or with both B02 and disulfiram (right) for 3 days, and cell growth was measured by MTA assay. Mean ± SD of quadruplicate experiments is shown.

BRCA1 and BRCA2 proteins are crucial mediators for assisting HR recombinase RAD51.^12^ Loss of *BRCA* function due to loss of heterozygosity (LOH), which invariably occurs in cancer‐initiating cells or cancer stem cells, drastically reduces HR function. These HR proteins are also needed to stabilize stalled replication forks in a manner independent of HR activities^16^ to prevent degradation upon replication stress, and to restart the fork for survival. If *ALDH2* function is reduced, higher levels of DNA damage or replication stress are provoked; therefore, combined loss of *BRCA1* or *BRCA2* and *ALDH2* are synthetically lethal. Indeed, it has been reported that *ALDH2* deletion or inhibition suppresses the proliferation of human or mice cells.^6^ We confirmed this experimental observation using ALDH2 inhibitor disulfiram and RAD51 inhibitor B02 in cancer cell lines HCT116 and MCF7 (Figure 3A,B).

## 4 DISCUSSION

Previous studies indicated that combined loss of *ALDH2* and *BRCA1/2* genes result in decreased survival of cells (synthetic lethality).^6^ Similarly, the *BRCA*‐related Fanconi anemia gene and *ALDH2* are required to sustain cell growth in leukemic cells.^7^ Another study concluded that the *ALDH2* variant may increase the levels of aldehydes that destabilize BRCA2 protein, accelerating genome instability and cancer initiation.^13^ These results strongly suggested that the ALDH2*2 genotype should affect breast cancer development. Our current study was planned to test this hypothesis. However, our data indicated that the impact of *ALDH2* rs671 on cancer development in Japanese HBOC patients are marginal, and the *ALDH2* variant neither prevents nor accelerates the initiation and progression of breast cancer.

Our observation and the experimental evidence mentioned above appeared to be a bit conflicting. This could mean that higher levels of endogenous DNA damage are generated in cultured cell lines than in the HBOC patient body (such as in cancer‐initiating breast epithelium), necessitating the interplay between *ALDH2* and HR in cell lines. Notably, how *ALDH2* contributes to reducing endogenous DNA damage in cell culture remains unclear. Potential sources of genome damage include not only acetaldehydes but also formaldehyde or other aldehydes. It is interesting to note that alcohol consumption was found to not be associated with breast cancer risk in HBOC women.^17^ Additionally, our recent discovery of a novel FA‐like IBMFS, aldehyde degradation deficiency syndrome (ADDS), has led to the notion that ALDH2 provides an important detoxication mechanism against formaldehyde.^18^, ^19^ Effects of oxygen radicals could also be mitigated by ALDH2 function. Given the current data, the genotoxins we discussed above may not exist at high concentrations in developing breast cancer cells. Another interpretation could be that *ALDH2* is not expressed in the early breast cancer tissues; however, this seems a remote possibility since ALDH2 is reported to be expressed in breast cancer stem cells.^20^

Our data have a few additional implications. First, our data did not support the view that *ALDH2* should be considered as a cancer therapeutic or prevention target in HBOC. For example, the use of disulfiram has been proposed for the treatment of cancers associated with *BRCA1/2* mutations.^6^ However, our data indicated that ALDH2 inhibitors may not be a good therapeutic measure for this purpose. Second, our data also did not support the view that the *ALDH2* variant accelerates cancer by destabilizing BRCA2.^13^ Experimental evidence suggests exogenous aldehyde reduces levels of BRCA2, not BRCA1, and the low levels of BRCA2 could induce genome instability and cancer.^13^ We did not detect a difference in age at diagnosis between *BRCA1* and *BRCA2* mutation carriers, and this also could mean that aldehydes do not significantly contribute to carcinogenesis in our patients.

In conclusion, our current study provides evidence for the lack of impact of the *ALDH2* rs671 on breast cancer development in HBOC patients. Our cross‐sectional design among a not necessarily large case‐dominant population did not allow us to evaluate the impact of *ALDH2* genotypes in a prospective manner. Despite this limitation, considering that *ALDH2* rs671 has a significant impact on aldehyde metabolism, the present results are sufficient to lead us to conclude that *ALDH*2 rs671 does not have a dramatic effect on the clinical presentation of HBOC.

## AUTHOR CONTRIBUTIONS

**Tomoharu Mori:** Data curation (lead); formal analysis (lead); investigation (lead); visualization (lead); writing – original draft (equal). **Yusuke Okamoto:** Data curation (lead); formal analysis (lead); investigation (lead); validation (lead); writing – original draft (equal). **Anfeng Mu:** Formal analysis (lead); investigation (lead); validation (equal); visualization (lead); writing – review and editing (equal). **Yoshimi Ide:** Data curation (equal). **Akiyo Yoshimura:** Data curation (equal). **Noriko Senda:** Data curation (equal). **Yukiko Inagaki‐Kawata:** Data curation (equal). **Masahiro Kawashima:** Data curation (equal). **Hiroyuki Kitao:** Data curation (equal). **Eriko Tokunaga:** Data curation (equal). **Yoshio Miyoshi:** Data curation (equal). **Shozo Ohsumi:** Data curation (equal). **Ko‐ichiro Tsugawa:** Data curation (equal). **Tomohiko Ohta:** Data curation (equal). **Toyomasa Katagiri:** Data curation (equal). **Shigeru Ohtsuru:** Project administration (equal). **Kaoru Koike:** Project administration (equal). **Seishi Ogawa:** Data curation (equal). **Masakazu Toi:** Data curation (equal). **Hiroji Iwata:** Data curation (equal). **Seigo Nakamura:** Data curation (equal). **Keitaro Matsuo:** Formal analysis (lead); investigation (equal); supervision (lead); validation (lead); writing – original draft (lead). **Minoru Takata:** Conceptualization (lead); data curation (equal); formal analysis (lead); funding acquisition (lead); investigation (lead); project administration (lead); supervision (lead); validation (lead); visualization (equal); writing – original draft (lead); writing – review and editing (lead).

## CONFLICT OF INTEREST

Drs. Toyomasa Katagiri, Seishi Ogawa, Masakazu Toi, Hiroji Iwata, and Keitaro Matsuo are editorial board members of Cancer Science. The authors declare that there is no conflict of interest except for: the lecture fee for Masakazu Toi (Eli Lilly, AstraZeneca, Daiichi‐Sankyo), Eriko Tokunaga (AstraZeneca), Yasuo Miyoshi (AstraZeneca), Tomohiko Ohta (AstraZeneca), and Shozo Ohsumi (AstraZeneca); the research funds for Masakazu Toi (Taiho, AstraZeneca, Kyowa‐Kirin, Shimadzu, Astellas, JBCRG association, AFI Technology, Yakult, Luxonus, GL Science, KBCRN association), and Hiroyuki Kitao (Taiho); the scholarship endowment for Masakazu Toi (Chugai, Eisai, Nippon Kayaku). Hiroyuki Kitao belongs to an endowed chair funded by Taiho and accepted researchers from Taiho.

## Supporting information


TableS1

TableS2

## Data Availability

The data that supports the findings of this study are available in the supplementary material of this article.
